# Prevalence and determinants of intestinal parasitic infections among immigrants in refugee camps in Tigray Region, Northern Ethiopia

**DOI:** 10.1186/s12879-026-14137-1

**Published:** 2026-08-05

**Authors:** Gebregiorgis Teklu, Ebud Ayele, Hailay Gebreyesus, Merhawi Alemu, Mulugeta Kiros, Letebrhan Hagos, Gebretsadkan Gebrekiristos, Tuemelsan Gebrewahis, Kiros Tedla, Gessessew Bugssa

**Affiliations:** 1 Medical Parasitology and Entomology Unit, Département of Medical Laboratory Science, College of Medicine and Health Sciences, Axum University, Axum, Ethiopia; 2 Department of Public Health Nutrition, College of Medicine and Health Science, Axum University, Axum, Ethiopia; 3 Department of Public Health, College of Health Science and Comprehensive Specialized Hospital, Axum University, Axum, Ethiopia; 4 Vector Biology and Immunology Unit, Department of Medical Laboratory Science, College of Medicine and Health Sciences, Medical Parasitology, Adigrat University, Adigrat, Ethiopia; 5 Unit of Medical Microbiology, Department of Medical Laboratory Science, College of Medicine and Health Sciences, Raya University, Raya University, Maychew, Ethiopia; 6 Unit of Medical Microbiology, Department of Medical Laboratory Science, College of Medicine and Health Sciences, Axum University, Axum, Ethiopia; 7 Département of Medical Laboratory Science, College of Medicine and Health Sciences, Medical Parasitology, Axum University, Axum, Ethiopia; 8 Faculty of Medical Laboratory Sciences, College of Health Sciences, Mekelle University, Mekelle, Ethiopia

**Keywords:** Intestinal parasites, Associated factors, Immigrants, Tigray, Ethiopia

## Abstract

**Background:**

Intestinal parasitic infections represent a significant health challenge globally. The World Health Organization recognizes that refugees are particularly susceptible to intestinal parasites. Refugee populations are especially vulnerable to intestinal parasitic infections due to unfavorable environmental and socioeconomic conditions, including overcrowding, inadequate sanitation, unsafe water supplies, and limited healthcare access. Nevertheless, epidemiological data on the prevalence and determinants of these infections among refugees in Ethiopia are limited especially within the Tigray refugee camps. This study aimed to determine the prevalence of intestinal parasites and determinant factors among immigrants in refugee camps of Tigray, northern Ethiopia.

**Method:**

A Community-based cross-sectional study was carried out among 422 selected participants using a systematic random sampling method from February to May 2020, in Shimelba and Hitsats refugee camps. Questionnaires and laboratory investigations were used to collect data. Stool samples were examined using wet mount and formol-ether concentration techniques based on standard procedures. Data were entered using Epi-data version 3.1 and analysed using SPSS version 25.0. Bivariable and multivariate logistic regression models were used to identify factors associated with intestinal parasitic infection. Variables with p-value ≤ 0.25 level of significance using bivariable logistic regression analysis were included in the multivariable logistic regression analysis. The significance level was declared at a p-value of < 0.05 in the final model and the model’s goodness-of-fit was assessed using the Hosmer–Lemeshow test.

**Result:**

The overall prevalence of protozoan parasites were 18.96% (80/422) and the prevalence of helminthes were 7.6% (32/422). The predominant parasite was *Giardia lamblia* (49) followed by *Entamoeba histolytica/dispar* (31), *H. nana* (12), *S. mansoni* (8), *A. lumbercoides* (5), Hookworm (3), and E. *vermicularis* (2). Absence of hand washing with soap before meals [AOR = 2.3; 95% CI (1.26, 4.36) dirty fingernails [AOR = 2.04; 95% CI (1.18, 3.53)], open field defecation [AOR = 2.1; 95% CI (1.12, 3.93)] and eating raw or poorly washed vegetables [AOR = 2.7; 95% CI (1.32, 5.34)] showed statistically significance with intestinal parasitic infections.

**Conclusion:**

Intestinal parasitic infections were moderate prevalent among immigrants according the WHO Intestinal parasite prevalence classification. The present study revealed that intestinal parasitic infections remain a significant public health problem among refugees residing in refugee camps. The overall prevalence of intestinal parasites was considerable, indicating ongoing transmission within the camp settings. Therefore, improving health education on hand washing practice before meals, avoiding open field defecation, cleanliness of fingernails, avoiding eating raw or poorly washed vegetables and assisting with water and sanitary facilities should be considered to help reduce the prevalence of intestinal parasites among immigrants. Tailored interventional studies are also recommended to identify the correlations between displacement and intestinal parasitic infections to improve the health conditions of immigrants in Ethiopia and in settings with the same problem.

**Clinical trail number:**

Not applicable.

**Supplementary Information:**

The online version contains supplementary material available at https://doi.org/10.1186/s12879-026-14137-1.

## Introduction

Intestinal parasitic infections (IPIs) are among the primary public health problems responsible for morbidity and mortality in endemic areas which are brought on by intestinal helminthes and protozoa [1]. According to the WHO’s 2023 an estimated 1.5 billion people individuals suffered illness due to these infections, and approximately 3.5 billion people were impacted. Especially in developing nations with low socioeconomic status and substandard living conditions [1–4].

An estimated 160 million international migrants are living in high-income countries at the moment, most of whom are from low- or middle-income countries. Human migration has many causes, most of which are social, political, and economic, as well as natural disasters and war [5]. There are 844,589 registered refugees and asylum-seekers in Ethiopia, making it the third-largest refugee-hosting nation in Africa. The vast majority come from Somalia, Eritrea, and South Sudan. The vast majority reside in 24 camps for refugees spread throughout five regional states (Afar Region, Benishangul-Gumuz Region, Gambella Region, Tigray Region) [6]. Intestinal parasites are very common in immigrant and refugee communities worldwide. An estimated 2 billion people globally are infected with helminthes [7, 8].

Soil-transmitted helminthes (STHs) and intestinal protozoa are responsible for a significant amount of morbidity, death in tropical and sub-tropical climates [9]. In sub-Saharan Africa (SSA), the protozoan parasites *Entamoeba histolytica* and *Giardia lamblia* are the most prevalent intestinal parasites and are responsible for significant morbidity and mortality. Moreover, soil-transmitted helminthes,

Particularly hookworm, *Ascaris lumbricoides (A. lumbricoides)*, and *Trichuris trichiura (T. trichiura)* are also known to inflict significant morbidities and mortalities [10–12].

Globally, *A. lumbricoides* infects about 1.2 billion people, whereas *T. trichiura* and hookworms cause infections in 795 million and 740 million people, respectively [13].

*G. lamblia* is a major intestinal protozoan parasite that infects more than 200 million people worldwide, with approximately 50,000 symptomatic cases reported annually. *E. histolytica*, another important protozoan parasite, causes around 50 million severe infections and an estimated 100,000 deaths each year [14].

People are infected by eating infective stages (eggs, cysts) or by the parasites’ larvae penetrating the skin through contaminated soil, water, and undercooked meat and/or vegetables [15]. In developing countries, the prevalence of intestinal parasitic infections is affected by numerous factors, including socio-demographic factors linked to poverty, lack of proper sanitation, and safe drinking water, unsafe disposal of human waste, limited healthcare services, and living in unfavorable environmental conditions [16].

These group of parasites have been identified as major global sources of disease and mortality. In endemic locations, they are among the most prevalent parasite illnesses in humans and have been linked to significant morbidity and financial loss [17, 18]. Iron-deficiency anemia, slowed growth, reduced academic achievement, weight loss, vitamin A deficiency, abdominal discomfort, indigestion, and various other physical and mental health issues are among the complications linked to IPIs [11, 19].According to WHO, the effect of these parasitic diseases is also more pronounced among refugees which are considered vulnerable segments of the society.

Ethiopia, one of the African nations, carries the higher rates of intestinal parasite infections. According to research carried out in Ethiopia, it is believed that one in eight people have hookworm, one quarter has *T. trichiura*, and one-third have *A.lumbricoides*. Thus, in SSA, Ethiopia has the second-highest prevalence of ascariasis, ranks third for hookworm, and fourth for trichuriasis [20]. Numerous studies have investigated the prevalence of intestinal parasitic infections (IPIs) and associated risk factors in Ethiopia including in Tigray. However, the majority of them focused mainly on prisons (inmate), school children, preschool children, hospitals, university cafeterias and food handlers. Studies on the prevalence of intestinal parasites and associated factors among immigrants in Ethiopia including in Tigray refugee camps are under investigated. Therefore, this study aimed to determine the magnitude of intestinal parasites and to identify the associated factors among the immigrant population in Tigray. Moreover, this study will serve as baseline data to inform policymakers to design strategies, implement prevention and control measures, and guide researchers to further investigate the effect of intestinal parasites in refugee camps [20, 21].

## Methods and materials

### Study area

The study was conducted in the refugee camps found in Tigray Regional State, Northern Ethiopia. Tigray is one of the eleven regions of Ethiopia located in the northern part of the country. In Tigray, there were four refugee camps under the Shire Operation camp are Adi Harush, Shimelba, Mai-Aini, and Hitsats. According to the 2018 United Nations Higher Commissioner for Refugees (UNHCR) 44,683 refugees were residing in these four identified camps. This region hosted the highest number of immigrants in the country. Most of the immigrants in the region were from Eritrea. Each refugee camp had one health center and one elementary school.

This study was conducted in the Hintsats and Shimelba camps. Hitsats Camp, situated approximately 40 km from Shire, was founded in 2013 G.C. following the influx of increased numbers of Eritrean refugees into Ethiopia. It is the newest and youngest of the so- called ‘Shire Camps’. It is located at the edge of Hitsats town, and the lack of a physical barrier between town and camp ensures a de facto integration between the two areas. Hitsats town is small but has experienced growth since the establishment of the camp in 2013. According to Hintsats refugees camp profile in 2018, the town hosted 11,000 refugees in 1333 permanent shelters built. Shimelba refugee camp: this camp was established in May 2004 G.C and hosted 13,965 refugees in 1901 permanent shelters during the study period [22]. The camp is located between Shire Sheraro and 408 km away from Mekelle, the capital of the region, and 12,229 km away from Addis Ababa, the capital of Ethiopia.

### Study design and populations

A community-based cross-sectional study design was considered to determine the prevalence of intestinal parasites and associated factors among immigrants in Tigray from February 2020 to May 2020 EC. All populations living in the Shire operation camps were the source of data for the study and the immigrant population residing in the Shimelba and Hitsats camps were the study population. Individuals (all age groups) who were willing to provide stool samples at the time of data collection were included in the study. On the other hand, those who were not available at the time of data collection and participants who were treated for intestinal parasitic infection in the last three weeks before the study were excluded.

### Sample size determination and sampling technique

#### Power calculation

The sample size was calculated using the single population proportion formula considering the following parameters and assumptions: Level of significance = 5%, the margin of error (d) = 5%. There is lack of published information about the prevalence of IPIs among immigrants in our country, we assumed 50% prevalence (*p* = 0.5) [23].$\:\mathbf{n}=\left(\frac{{\left(\mathbf{Z}\frac{\boldsymbol{\upalpha\:}}{2}\right)}^{2}\mathrm{*}\mathbf{p}(1-\mathbf{P})}{{\mathbf{d}}^{2}}\right)$

Considering a 10% non-response rate, it gives a final sample size of 384 + 38=422. Hence, a total of 422 participants were included in the study.

#### Sampling technique

Among the four Refugee Camps in the region, two refugee camps were randomly selected for the study. Since the immigrants are registered in a log book, a systematic random sampling method was used to select the study participants. To determine the sampling interval, the total number of immigrants registered in the log book in that specific camp was divided by the proportionally allocated number of immigrants to be sampled from that specific camp ($\:\frac{Nj}{nj}$=k) which gives the **K**^th^ value, the interval in which we select the target participant every **K**^th^ time. If the selected participant was not eligible, the next participant was considered and continued until the desired sample size was saturated. A target number of participants per camp was proportionally allocated based on the number of immigrants in each camp. Once participants fulfilled the eligibility criteria, they were approached for their temporary housing through health personnel assigned in that camp and were invited to the nearby temporary sample collection area. The proportional allocation of participants to each camp was calculated as follows:

$$nj\, = \>{n \over N} \times \>Nj$$

nj is the sample size of the jth stratum.

Nj is the population size of the jth stratum.

n=n1 + n2+…+n is the total sample size.

N= N1 + N2+…+N is the total population size.

$$\eqalign{ & Shimelba{\rm{ }}Camp{\rm{ }}\left( {n{\rm{ }}1} \right){\rm{ }} = \cr & \>{{422\left( n \right)} \over {24965(N1 + N2)}} \times \>13965\left( {N1} \right) = 236\,then \cr}$$

k = $$\:\frac{Nj}{nj}$$

k = $\:\frac{13965}{236}=59$ then every 59th was selected

Hitsats camp (n2) =$\:\frac{422\left(n\right)}{24965(N1+N2)}\times\:11000\left(N2\right)=186$ then.

k = $$\:\frac{\boldsymbol{N}\boldsymbol{j}}{\boldsymbol{n}\boldsymbol{j}}$$

**k =** $$\:\frac{11000}{186}=59$$ was selected

### Data collection and study variables

Intestinal parasitic infection was the dependent variable of this study and sociodemographic factors (Age, Sex, Educational level, Family size, Occupation and Monthly income.) and Behavioral factors (Eating uncooked/raw meat, Water source for drinking, Trimming fingernails, Eating raw/poorly washed vegetables, Place of defecation, Shoes wearing habit, Cleanliness of fingernails, Hand washing practice before meal with soap, Habit of hand washing after using toilet, Washing clothes in reservoir /water body, Source of water for bathing and Animal contact) were the independent variables. Socio-demographic and behavioral information was gathered using a structured questionnaire administered by interviewers, along with an observational checklist. This helps the data collector not to miss any of the important topics under consideration. The Questionnaire was developed after reviewing relevant literature and WHO guideline.

### Laboratory diagnosis

#### Parasitological examination

**Stool collection** The selected individuals for the study were supplied with labeled, clean plastic cups with applicator sticks to bring approximately 2 g of fresh stool specimens. All stool samples were collected on-site at a designated central location.

Direct wet mount preparation: A small sample of stool (approximately 2 mg) was analyzed using a direct wet mount prepared with a drop of 0.85% saline solution. And then systematically examined with the low power objective (10x) so that the entire coverslip area was scanned for the presence of parasite stages (trophozoite/cysts/oocyst) and examined with the high-power objective lens (40x) to see more detailed morphology of the object for confirmation. The leftover portion was stored in 10% formalin at a ratio of 1 gram of stool to 5 milliliters of formalin, allowing for repeat testing when needed. Formol-ether concentration (FEC) technique: enables the detection of small numbers of organisms that may be missed by using wet mount. Sedimentation methods were used to concentrate protozoa, helminthic ova, and larvae in the bottom of the tube. FEC was done in the refugee clinic. In the FEC procedure, approximately 0.5 g of feces was mixed with 10 ml of Normal saline and the mixed stool was strained via gauze into a funnel. The strained contents were collected into 15 ml conical centrifuge tube. About 2.5 ml of 10% formalin and 1 ml of diethyl ether were then added and centrifuged for 1 min at 3000 rpm. The supernatant was discarded and a drop of the sediment was covered with microscopic slides and then examined using low power objective lens (10x) and high-power objective lens (40x) to see a more detailed morphology of the object for confirmation [24].

### Data quality assurance and management

Training was provided to the data collectors. The questionnaire was initially developed in English and subsequently translated into Tigrigna, the local language of the study area. A pretest of the questionnaire was conducted on 5% of the sample in Mai- Aini refugee camp. All reagents were inspected for expiration dates. The principal investigator closely supervised the entire process.

Laboratory examinations were performed in accordance with standard operating procedures (SOPs). Prior to commencing the work, the laboratory equipment was inspected to ensure it was functioning correctly and handling according to the manufacturer’s standard procedures. Besides, reagents were checked for expiry dates. Fecal specimens were collected and processed in strict accordance with standardized procedures. The collected data were checked daily for consistency and accuracy.

### Data analysis

The data were reviewed for completeness, entered into EpiData version 3.0, and then exported to SPSS version 25 for analysis. The socio-demographic characteristics of the study population were summarized using frequencies, mean and standard deviation. An internal comparison was made using binary logistic regression to determine the independent effect of the variables by calculating the strength of the association between IPIs and associated factors using odds ratio (OR) at a 95% confidence interval (CI). For adjusting the confounding variables, variables with less than or equal to 0.25 level of significance using bivariable logistic regression analysis were included in the multivariate logistic regression analysis [25]. Significance values were considered whenever the p-value is < 0.05. And the model’s goodness of fit was assessed using the Hosmer–Lemeshow test.

### Ethical consideration

Ethical clearance was obtained from the Ethical Review Board of the College of Health Sciences, Mekelle University with reference number: ERC1540/2020. Clients were informed about the objective and purpose of the study and verbal and written informed consent was obtained from each participant. Parents of children < 18 years old were informed of their children’s participation and gave informed written consent for their children to participate. Assents were also collected from children. Clients’ information was kept confidential during the research and then after. Clients identified as positive for intestinal parasite infection were treated according to the national protocol.

## Results

### Socio- demographic characteristics of study participants

A total of 422 participants were successfully interviewed, and examined achieving a 100% response rate. Of the study participants, 214(50.71%) were females and 208 (49.28%) were males. The age of the study participants ranged from 2 to 82 years with a mean age of 25.9 (± SD) (25.49 ± 14.516). The majority (75.6%, 319/422) belonged to the age group of > 15 years. Among the study participant,8.5% (36/422) had a family size greater or equal to five and 91.5% (386/422) had a family size of fewer than five (Table 1).

**Table 1:** Demographic characteristics of immigrants among refugees in Tigray, Northern Ethiopia, 2020 (*N* = 422)

Variables	Categories	Frequency	Percentage (%)	Positive to IPI, *n* (%)
Age	< 5	23	5.5	10(43.5%)
	5–15	80	19.0	15(18.8%)
	> 15	319	75.6	87(27.3%)
Sex	Male	208	49.3	54(26.0%)
	Female	214	50.7	58(27.1%)
Educational level	No formal education	221	52.4	66(29.9%)
	Primary	161	38.2	35(21.7%)
	Secondary	30	7.1	8(26.7%)
	Diploma and above	10	2.4	3(30%)
Occupation	Farmer	35	8.3	13(37.1%)
	Merchant	33	7.8	6(18.2%)
	No occupation	354	83.8	93(26.2%)
Family size	< 5	386	91.5	99(25.6%)
	≥ 5	36	8.5	13(36.1%)
Monthly income in birr	< 500	338	80.1	85(25.1)
	500–2000	70	16.6	21(30.0%)
	> 2000	14	3.3	6(42.9%)

### Prevalence of intestinal parasites

The overall prevalence of intestinal parasites was found to be 26.5% (112/422). The overall prevalence of protozoa were 18.96% (80/422) and the prevalence of helminths were 7% (30/422). Of the 112 infected individuals, 98.2% (110/112) harbored a single parasite and 1.8% (2/112) harbored more than one parasite. The most prevalent intestinal parasite was *Giardia lamblia 11.6% (49/422)*, followed by *E. histolytica/dispar* 7.4% (31/422), *H. nana* 2.8% (12/422) and *S. mansoni* 2.0% (8/422). Single and double infections were identified from 110 (98.2%) and 2 (1.8%) study participants respectively. The prevalence of protozoan, helminthic and mixed infections were 19.0% (80/422), 7.1% (30/422) and 0.5% (2/422) respectively (Table 2).

**Table 2:** Distribution of intestinal parasites among immigrants in Tigray, Northern Ethiopia, 2020(*N* = 422)

Type of parasite	Frequency	Percentage	95% CI (%)
*Giardia lamblia*	49/422	11.6	8.9–15.0
*Entamoeba histolytica/dispar*	31/422	7.3	5.2–10.2
*Hymenolepis nana*	12/422	2.8	1.6–4.9
*Schistosoma mansoni*	8/422	1.9	1.0-3.7
*Ascaris lumbricoides*	5/422	1.2	0.5–2.7
Hookworm	3/422	0.7	0.2–2.1
*Enterobius Vermicularis*	2/422	0.5	0.1–1.7
Double parasitic infection			
*Schistosoma mansoni* and *Giardia lamblia*	1/422	0.2	0.04–1.33
*Hymenolepis nana* and *Entamoeba histolytica/dispar*	1/422	0.2	0.04–1.33
Protozoa	80/422	18.9	
Helminthes	30/422	7	
Mixed infection	2/422	0.5	

### Prevalence of intestinal parasites about the different risk factors

Out of the 422 participants, 38% (161/422) of them practiced hand washing before meal, and 81% (343/422) of them used latrine. 30% (95/314) of the participants who had a habit of eating raw unwashed or poorly washed vegeTable 30% (95/314) were positive for parasitic infection (Table 3).

**Table 3:** Prevalence of intestinal parasites about different behavioral factors among immigrants in the refugee of Tigray, northern Ethiopia, 2020

Variables	Categories	Frequency	Percent	Positive to IPI, *n* (%)	x2	*p*-value	
Place of defecation	Open field	79	18.7	36(45.6%)	16.87	0.001	
	Latrine	343	81.3	76(22.2%)			
Eating raw or poorly washed vegetables	Yes	314	74.4	95(30.3%)	7.95	0.005	
	No	108	25.6	17(15.75%)			
Source of water for drinking	Private tap water	35	8.3	9(25.7%)	6.33	0.042	
	Public tap water	370	87.7	94(25.4%)			
	Well	17	4.0	9(52.9%)			
Eating raw/under cooked meat	Yes	16	3.8	6(37.5%)	0.52	0.469	
	No	406	96.2	106(26.1%)			
Cleanliness of finger nails	Yes	312	73.9	67(21.5%)	14.78	0.001	
	No	110	26.1	45(40.9%)			
The habit of hand Washing with soap before meal	Yes	161	38.2	22(13.7%	21.08	0.001	
	No	261	61.8	90(34.5%)			
Animal contact	Yes	81	19.2	30(37.0%)	5.02	0.025	
	No	341	80.8	82(24.0%)			
Shoes Wearing habit	Sometimes	47	11.1	21(44.7%)	7.91	0.005	
	Always	375	88.9	91(24.3%)			
washing clothes in reservoirs/water body	Yes	24	5.7	13(54.2%)	8.52	0.004	
	No	398	94.3	99(24.9%)			
Place for bathing	At home	35	8.3	12(34.3%)	6.77	0.034	
	At reservoir	18	4.3	9(50.0%)			
	Public bathroom	368	87.4	91(24.7%)			
Habit of hand washing after toilet	Yes	224	53.1	37(16.5%)	23.51	0.001	
	No	198	46.9	75(37.9%)			
trimming your fingernail	Yes	40	9.5	17(42.5%)	4.9	0.001	
	No	382	90.5	95(24.9%)			

There was no significant difference in prevalence of the IPIs between male and females. Similarly, although there was no statistically significant association between age and parasitic infection, the highest parasite prevalence was reported among the age group of > 15 years (*P* > 0.05).

### Factors associated with intestinal parasites

Behavioral and sociodemographic factors were assessed for association with intestinal parasitic infections among the study participants. These factors included Age, place of defecation, eating raw/poorly washed vegetable, Cleanliness of fingernails, Habit of hand washing with soap before meal, animal contact, Source for drinking water, Shoe wearing habit, place for bathing, Habit of hand washing after toilet, trimming finger nails, Family size and washing clothes in reservoirs/water body.

The distribution of intestinal parasitic infections according to different behavioral factors is presented in (Table 4).

**Table 4:** Bivariable logistic regression analysis of factors associated with intestinal parasitic infections among refugees (*N* = 422)

Variables	Categories	IP		COR (95%CI)	P-value
		Presence (%)	Absence (%)		
Age	< 5	10(43.5)	13(56.5)	2.11(0.87-4.85)	0.101
	5–15	15(18.8)	65(81.3)	0.62(0.33-1.14)	0.124
	> 15	87(27.3)	232(72.7)	1	
Place of defecation	Open field	36(45.6)	43(54.4)	2.94(1.77–4.90)	0.001
	Latrine	76(22.2)	267(77.8)	1	
Eating raw/poorly washed vegetable	Yes	95(30.3)	219(69.7)	2.32(1.31–4.11)	0.004
	No	17(15.7)	91(84.3)	1	
Cleanliness of fingernails	Yes	67(21.5)	245(78.5)	1	
	No	45(40.9)	65(59.1)	2.53(1.59–4.04)	0.001
Habit of hand washing with soap before meal	Yes	22(13.7)	139(86.3)	1	
	No	90(34.5)	171(65.5)	3.33(1.98–5.58)	0.001
animal contact	Yes	30(37.0)	51(63.0)	1.86(1.11–3.11)	0.018
	No	82(24.0)	259(76.0)	1	
Source for drinking water	private tap water	9(25.7)	26(74.3)	1	
	Public tap water	94(25.4)	276(74.6)	0.98(0.45-2.18)	0.960
	Well	9(52.9)	8(47.1)	3.25(0.96-10.98)	0.058
Shoe wearing habit	Sometimes	21(44.7)	26(55.3)	2.52(1.35,0.4.69)	0.004
	Always	91(24.3)	284(75.7)	1	
place for bathing	At home	12(34.3)	23(65.7)	1	
	At reservoir	9(50.0)	9(50.0)	1.92(0.60 − 6.10)	0.268
	Public bathroom	91(24.7)	277(75.3)	0.63(0.30-1.32)	0.221
Habit of hand washing after toilet	Yes	37(16.5)	187(83.5)	1	
	No	75(37.9)	123(62.1)	3.08(1.96–4.86)	0.001
trimming finger nails	Yes	17(42.5)	23(57.5)	2.23(1.14–4.36)	0.019
	No	95(24.9)	287(75.1)	1	
Family size	< 5	99(25.6)	287(74.4)	1	
	≥ 5	13(36.1)	23(63.9)	1.64(0.80-3.36)	0.178
washing clothes in reservoirs/water body	Yes	13(54.2)	11(45.8)	3.57(1.55–8.22)	0.003
	No	99(24.9)	299(75)	1	

### Factors associated with intestinal parasites

The results of multivariate logistic regression analysis showed that use of open field defecation (AOR: 2.09, 95% CI (1.117, 3.925), absence of hand washing with soap before meal (AOR: 2.341, 95% CI (1.257, 4.358) and dirty fingernails (AOR: 2.039, 95% CI (1.179, 3.525) and habit of eating raw or poorly washed vegetables (AOR: 2.654, 95% CI (1.320, 5.337) were significantly associated with IPIs. (Table 5).

**Table 5:** Multivariable logistic regression analysis of factors associated with intestinal parasitic infections among refugees (*N* = 422)

Variables	Categories	IP		AOR (95%CI)	P-value
		Presence (%)	Absence (%)		
Age	< 5	10(43.5)	13(56.5)	1.27(0.41–3.88)	0.678
	5–15	15(18.8)	65(81.3)	0.58(0.29-1.15)	0.116
	> 15	87(27.3)	232(72.7)	1	
Place of defecation	Open field	36(45.6)	43(54.4)	2.09(1.12–3.93)	0.021*
	Latrine	76(22.2)	267(77.8)	1	
Eating raw/poorly washed vegetable	Yes	95(30.3)	219(69.7)	2.65(1.32–5.34)	0.006*
	No	17(15.7)	91(84.3)	1	
Cleanliness of fingernails	Yes	67(21.5)	245(78.5)	1	
	No	45(40.9)	65(59.1)	2.04(1.18–3.53)	0.011*
Habit of hand washing with soap before meal	Yes	22(13.7)	139(86.3)	1	
	No	90(34.5)	171(65.5)	2.34(1.26–4.36)	0.007*
animal contact	Yes	30(37.0)	51(63.0)	1.31(0.66-2.63)	0.442
	No	82(24.0)	259(76.0)	1	
Source for drinking water	private tap water	9(25.7)	26(74.3)	1	
	Public tap water	94(25.4)	276(74.6)	0.72(0.26-1.99)	0.528
	Well	9(52.9)	8(47.1)	1.78(0.42-7.56)	0.433
Shoe wearing habit	Sometimes	21(44.7)	26(55.3)	0.942(0.42-2.13)	0.886
	Always	91(24.3)	284(75.7)	1	
place for bathing	At home	12(34.3)	23(65.7)	1	
	At reservoir	9(50.0)	9(50.0)	0.452(0.09-2.39)	0.35
	Public bathroom	91(24.7)	277(75.3)	0.762(0.28-2.09)	0.599
Habit of hand washing after toilet	Yes	37(16.5)	187(83.5)	1	
	No	75(37.9)	123(62.1)	1.62(0.92-2.86)	0.096
trimming finger nails	Yes	17(42.5)	23(57.5)	1.61(0.72-3.62)	0.249
	No	95(24.9)	287(75.1)	1	
Family size	< 5	99(25.6)	287(74.4)	1	
	≥ 5	13(36.1)	23(63.9)	1.211(0.51-2.89)	0.665
washing clothes in reservoirs/water body	Yes	13(54.2)	11(45.8)	3.35(0.98-11.47)	0.055
	No	99(24.9)	299(75)	1	

## Discussions

The result of this study showed that 26.5% of the participants were infected with intestinal parasites. The finding was similar to community-based studies from different parts of Ethiopia. such as Gonder (24.7%) [26], in Adama Town (21.4%) [27], and other countries, such as refugees in Texas (25.3%) [28], Gaza Strip (24.6%) [29], and Nuseirat refugee camp, Greece (29.8%) [30].However, it is relatively higher than studies reported from Kiryandongo refugee camp, Uganda (8%) [31], Durban refugee camp in South Africa at (18.8%) [32], California refugee (15%) [33], German refugee (17%) [34], among asylumseekers in Italy (20.6%) [35], among immigrants in Rome (15%) [36], and Quatar (18.7%) [37], the observed discrepancy might be due to differences in climatic condition of study area, geographic locations, individual behavioral habits and socio-economic of the study participants.

However, the findings of this study were lower than those reported in other community-based studies. The studies include Southwest Ethiopia (83%) [38], Awramba (44.1%) [26], Somali refugee (38%) [39], Nigeria refugee (79.5%) [40], and Kentucky refugee (51%) [41]. the difference might be due to the methods employed for stool examination, sample size taken, and study period difference.

The predominant parasite identified in the present study was *Giardia lamblia* with a prevalence of 11.6%. This is similar to the study done in refugees in Bangladesh (9.5%) [42]. This is higher than the study done in Uganda refugees (2.9%) [30], and California refugees (4.1%) [33], in Rome immigrants (3.64%) [35]. But lower than the study done in the Afghan refugee population which was found to be 33.3% [43]. This might be due to differences in sample size and study period. The high prevalence of this parasite may be attributed to its easy transmission, as it is commonly present in contaminated food, water, soil, or surfaces exposed to fecal matter.

The second dominant intestinal parasite detected was *E. histolytica/dispar* with the prevalence of 7.3%. This was in line with a prevalence 8.7% done in Awramba [44]. This was, however, higher than studies done in refugee camps of Nigeria, and Gaza which reported prevalence of 6.2% and 5.6%, respectively [40, 45]. On the other hand, the findings were lower than Studies conducted in Arbaminch town (11.4%) [15], El-Fashir refugee camp, north -Darfur (16.1%) [46], and Nepal (11.9%) [47]. The variations in the prevalence of *E. histolytica/dispar* reported across studies may be attributed to differences in drinking water contamination levels, the practice of consuming raw vegetables, and the hand-washing behaviors of the study participants. Differences in hand-washing practices among study participants, consumption of raw vegetables, and levels of environmental contamination across localities lead to variations in the prevalence of amoebic infection between studies.

Moreover, this study indicated that individuals who used open field defecation were 2 times (AOR: 2.09, 95% CI (1.117, 3.925) more likely to be infected with intestinal parasites than those who used latrine. This might be due to contamination of soil with human waste during open field defecation they can easily harbored to intestinal parasites as they ingest food and during defecation by penetration of larvae. Similar findings were reported by Southwest Ethiopia [48].

Those who hadn’t the habit of washing hands with soap before meals were 2 times (AOR: 2.341, 95% CI (1.257, 4.358) more likely to be infected with intestinal parasites as compared to those who had the habit of washing hands with soap before meals. why because it is conducive environment for faecal-oral transmission of intestinal parasites. Similar findings were reported by others [40]. It might be due dirty particles remain in their hand and contaminated as they ingest food and limited habit of use of soap for cleaning hands before meal.

Individuals who had the habit of eating raw or poorly washed vegetables showed a 2 times higher prevalence of intestinal parasitic infection than those who didn’t eat raw or poorly washed vegetables and the difference was statistically significant (AOR: 2.654, 95% CI (1.320,5.337). Similar findings were reported by others [33]. This might be due to the unwashed or undercooked vegetables contaminated with fecal materials on the farm and contamination of the vegetables with wastes in the home creating a conducive environment for the transmission of intestinal parasitic infections. But contrary to this study, the study conducted by Ethiopian Gonder, northwest Ethiopia reported of no association between eating raw or poorly washed vegetables with intestinal parasitic infections [49]. This difference might be due to the differences in environmental sanitation between the two study areas.

To this end, individuals with dirty fingernails were 2 times (AOR: 2.039, 95% CI (1.179, 3.525) more likely to be infected with intestinal parasites than those with clean fingernails. Similar findings were reported elsewhere [50]. This could be explained by the fact that parasites may remain under fingernails with soil and causing infections when people ingest food.

### Limitations of the study

The study is not free of limitations. Due to resource constraints, the study was not able to include or perform other sensitive methods such as Kato-Katz and PCR. The study also shares the limitations of the cross-sectional study design.

### Conclusions and recommendations

The overall prevalence of intestinal parasitic infection among immigrants in refugee camps of Tigray was high. The prevalence of infection was higher for protozoan parasites compared to helminthes. The study also identified dirty fingernails, open field defecation, habit of eating raw or poorly washed vegetables and absence of hand washing habit with soap before meals were significantly associated with intestinal parasitic infection. Improved health education on hand washing practice before meals, avoiding open field defecation, cleanliness of fingernails and avoiding eating raw or poorly washed vegetables should be taken into account to reduce the prevalence of intestinal parasites. Building enough toilets, and health facilities expansion, providing safe water for drinking and giving attention to deworming programs would reduce the prevalence of intestinal parasites in the refugees. Tailored interventional studies are also recommended to identify the correlations between displacement and intestinal parasitic infections to improve the health conditions of immigrants in Ethiopia and in settings with the same problem. Additionally, research based on advanced parasitological and molecular tests like PCR and whole genome sequencing are also recommended to illustrate the true burden of Intestinal parasites and to identify between species of parasites.

## Supplementary Information

Below is the link to the electronic supplementary material.

Supplementary Material 1

## Data Availability

No datasets were generated or analysed during the current study.

## Declarations

### Assent

Informed, written, and signed consent was obtained from Parent’s/guardian’s after the purpose and benefits of the study were discussed. Parent’s/guardian’s were informed about the minimal risk they had taken part in the study subject, their volunteerism, and the right to leave the interview and producers at any time they wanted. Confidentiality and information of the study participant will also be kept secure.

## Abbreviations

BD: Bowel Disease
IPIs: Intestinal Parasite Infections
SOPs: Standard Operating Procedures
SPSS: Statistical Product and Service Solutions
UC: Ulcerative Colitis
CSA: Central Statistical Agency
AOR: Adjusted Odds Ratio
COR: Crude Odds Ratio
CI: Confident Interval
