# Supplementary material for: Prevalence and determinants of intestinal parasitic infections among immigrants in refugee camps in Tigray Region, Northern Ethiopia
Source: BMC Infect Dis. 2026 Aug 5;26:1927. doi: 10.1186/s12879-026-14137-1 (PMC13729656; doi:10.1186/s12879-026-14137-1)
Supplement: Supplementary file 1 [file 12879_2026_14137_MOESM1_ESM.docx]

Questionnaire to be filled

Patient code No (given by the principal investigator) __________________

Part 1:-

Socio demographic data:

1. Age---------

2. Sex: A.Male	B. Female

3. Educational level: A. No formal education B. Primary C. Secondary D. College and above

4. What is your reliogion A.Orthodox B.Muslim C. Protestant D. Catholic

5. Occupation A. Farmer B. Merchant C. Government employee D. No occupation

6 .Family size ………..

7. Monthly income……….

Part 2:-behavioral data

1. Place of defecation A. Open filed	B. Latrine

2. Eating raw or poorly washed vegetable A. Yes B. No

3. Source of water for drinking A. Private tap water B. Public tap water C. Well

4. Eating raw/under cooked meat A.Yes B.No

5. Cleanness of finger nails A.Yes B.No

6. Washing hand with soap before meal A.Yes B.No

7 Animal contact A.Yes B.No

8. Shoes Wearing habit A. Sometimes B. Always

9. Washing clothes in reservoirs/water body B Yes B. No

10 Place for bathing A. At home B. At reservoir C. Public bath room

11. Hand washing habit after toilet A.Yes B.No

12. Trimming your finger nail A.Yes B. No

**Observational checklist**

|  | YES | NO |
| --- | --- | --- |
| Cleanness of finger nails |  |  |
| Habit of trimming finger nails |  |  |
| Habit of wearing shoe |  |  |
| Place of defecation |  |  |
